# Supplementary material for: Exploring ligand binding pathways on proteins using hypersound-accelerated molecular dynamics
Source: Nat Commun. 2021 May 14;12:2793. doi: 10.1038/s41467-021-23157-1 (PMC8121818; doi:10.1038/s41467-021-23157-1)
Supplement: Supplementary file 1 — Supplementary Information [file 41467_2021_23157_MOESM1_ESM.pdf]

<Supplementary Information>

Exploring ligand binding pathways on proteins using hypersound–accelerated molecular dynamics

Mitsugu Araki[1,*], Shigeyuki Matsumoto[2], Gert-Jan Bekker[3], Yuta Isaka[4], Yukari Sagae[1], Narutoshi Kamiya[5], and Yasushi Okuno[1,2,*]

[1] Graduate School of Medicine, Kyoto University, 53 Shogoin-Kawaharacho, Sakyo-ku, Kyoto 606-8507, Japan

[2] Medical Sciences Innovation Hub Program, RIKEN Cluster for Science, Technology and Innovation Hub, 1-7-22 Suehiro-cho, Tsurumi-ku, Yokohama City, Kanagawa 230-0045, Japan

[3] Institute for Protein Research, Osaka University, 3-2 Yamadaoka, Suita, Osaka 565-0871, Japan

[4] Research and Development Group for *In Silico* Drug Discovery, Center for Cluster Development and Coordination (CCD), Foundation for Biomedical Research and Innovation at Kobe (FBRI), 6-3-5, Minatojima-minamimachi, Chuo-ku, Kobe, Hyogo 650-0047, Japan

[5] Graduate School of Simulation Studies, University of Hyogo, 7-1-28 Minatojima-minamimachi, Chuo-ku, Kobe, Hyogo 650-0047, Japan

***Corresponding authors:**

Yasushi Okuno
Graduate School of Medicine, Kyoto University, 53 Shogoin-Kawaharacho, Sakyo-ku, Kyoto 606-8507, Japan. Phone: +81-75-751-3920, Email: okuno.yasushi.4c@kyoto-u.ac.jp

Mitsugu Araki
Graduate School of Medicine, Kyoto University, 53 Shogoin-Kawaharacho, Sakyo-ku, Kyoto 606-8507, Japan. Phone: +81-75-751-3920, Email: araki.mitsugu.6w@kyoto-u.ac.jp

Contents

1 Supplementary Figures ······ 3
2 Supplementary Tables ······ 20
3 Supplementary References ······ 24

1. Supplementary Figures

Supplementary Figure 1

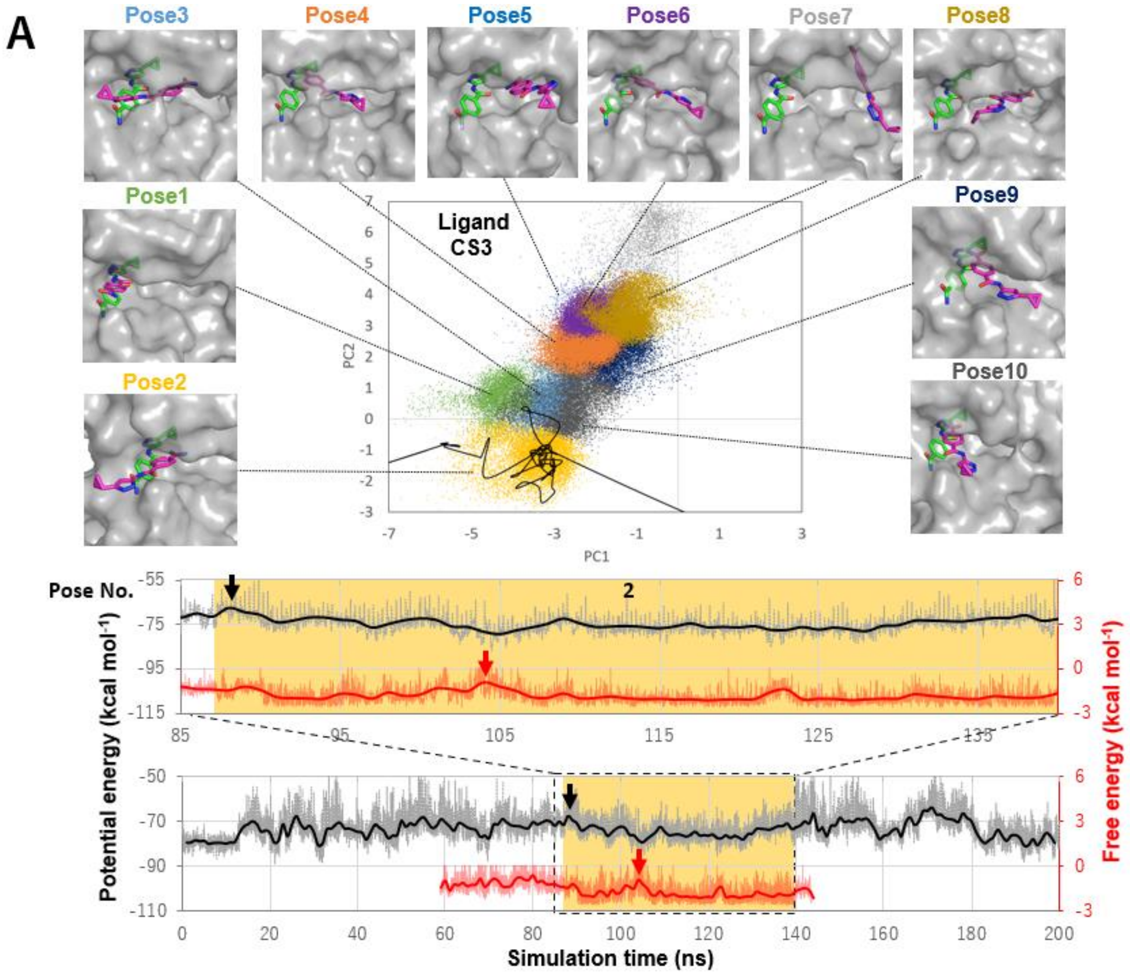

Supplementary Figure 1 (Continued)

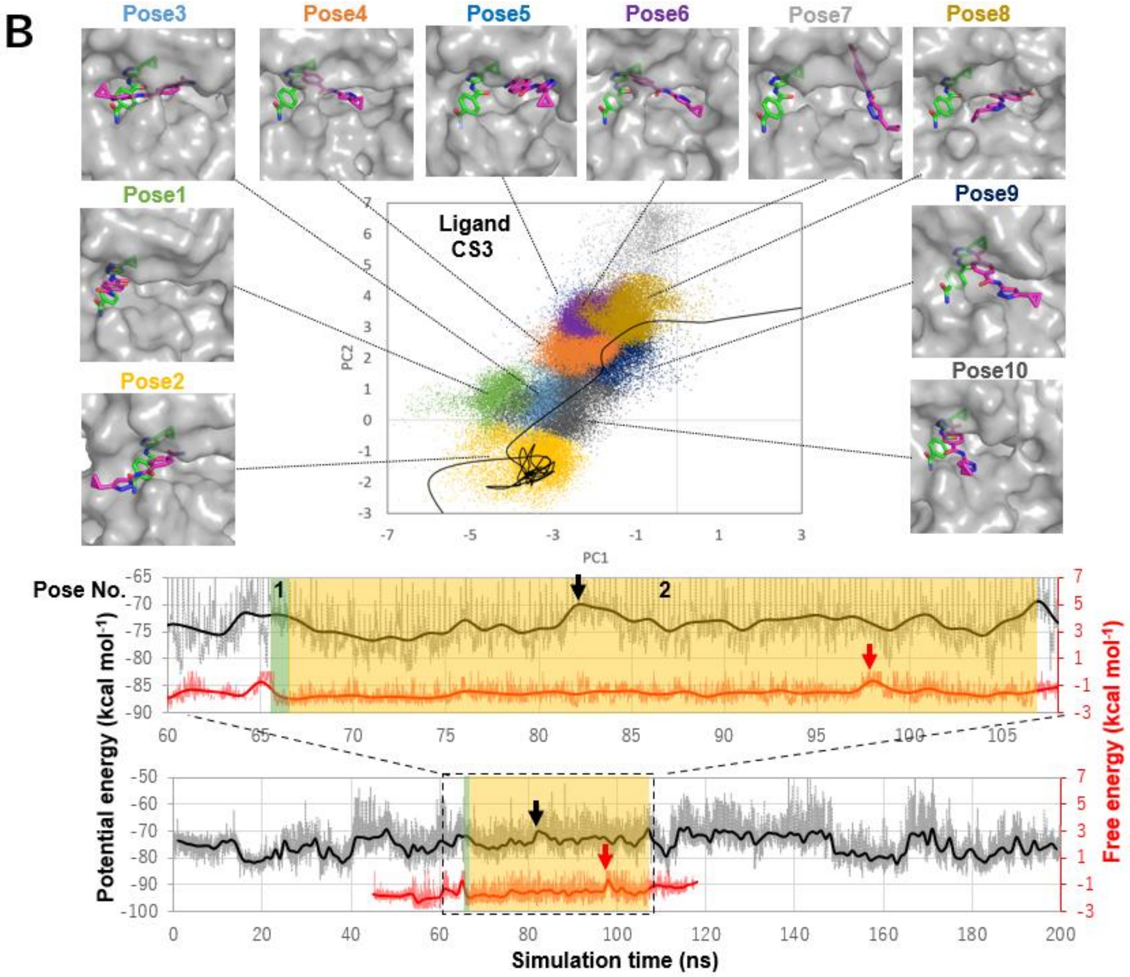

Supplementary Figure 1 (Continued)

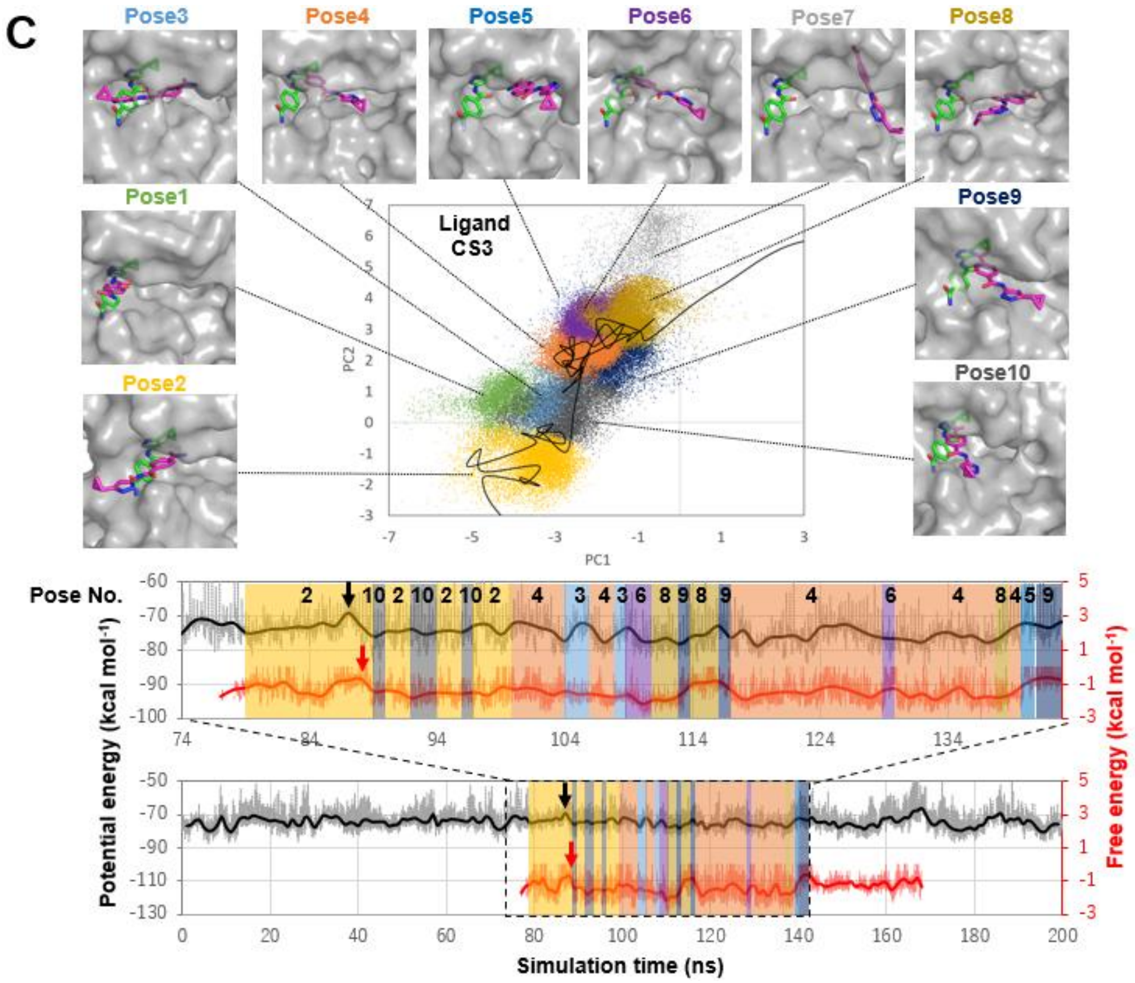

Supplementary Figure 1. Three representative binding pathways of the CS3 ligand to the ATP-binding pocket of CDK2. In pathway (A), the transition state occurs upon entry into the CDK2 pocket; in (B), the transition state is reached during conformational rearrangement in the pocket interior, whereas both ligand binding and unbinding are observed in pathway (C). (top) Projections of binding conformations observed in the whole set of MD trajectories (colored dots) and of a representative binding pathway (black line) onto the first and second principal components (PC1 and PC2) calculated from PCA (see Methods for details). Ten representative binding poses (magenta sticks)

on CDK2 (gray surfaces) are shown along with the crystallographic pose (green sticks), the closest conformation to which was designated as Pose 1. (bottom) Potential energy (black) and free energy (red) trajectories corresponding to the pathway shown in the PCA map. The potential energy was calculated as the sum of the intraligand and intermolecular (protein-ligand and ligand-solvent) contributions. The free energy trajectory was produced from the free energy landscape with respect to (PC1, PC2, and PC3) (Supplementary Fig. 6). The highest-energy transition state is indicated by a black (potential energy) or red (free energy) arrow. An enlarged view of these trajectories close to the highest-energy transition state is also shown in the panel above the whole trajectories. Time intervals in which ligand binding was observed are highlighted in the same color as that used for the binding conformation in the top panel. In 5 out of the 9 binding pathways observed in the hypersound-perturbed MD simulations with N = 50 steps, v_{max} = 400 m/s, and T_{int} = 2,400 N, the binding pose assigned to the highest-energy transition state is the same between potential energy and free energy trajectories.

Supplementary Figure 2

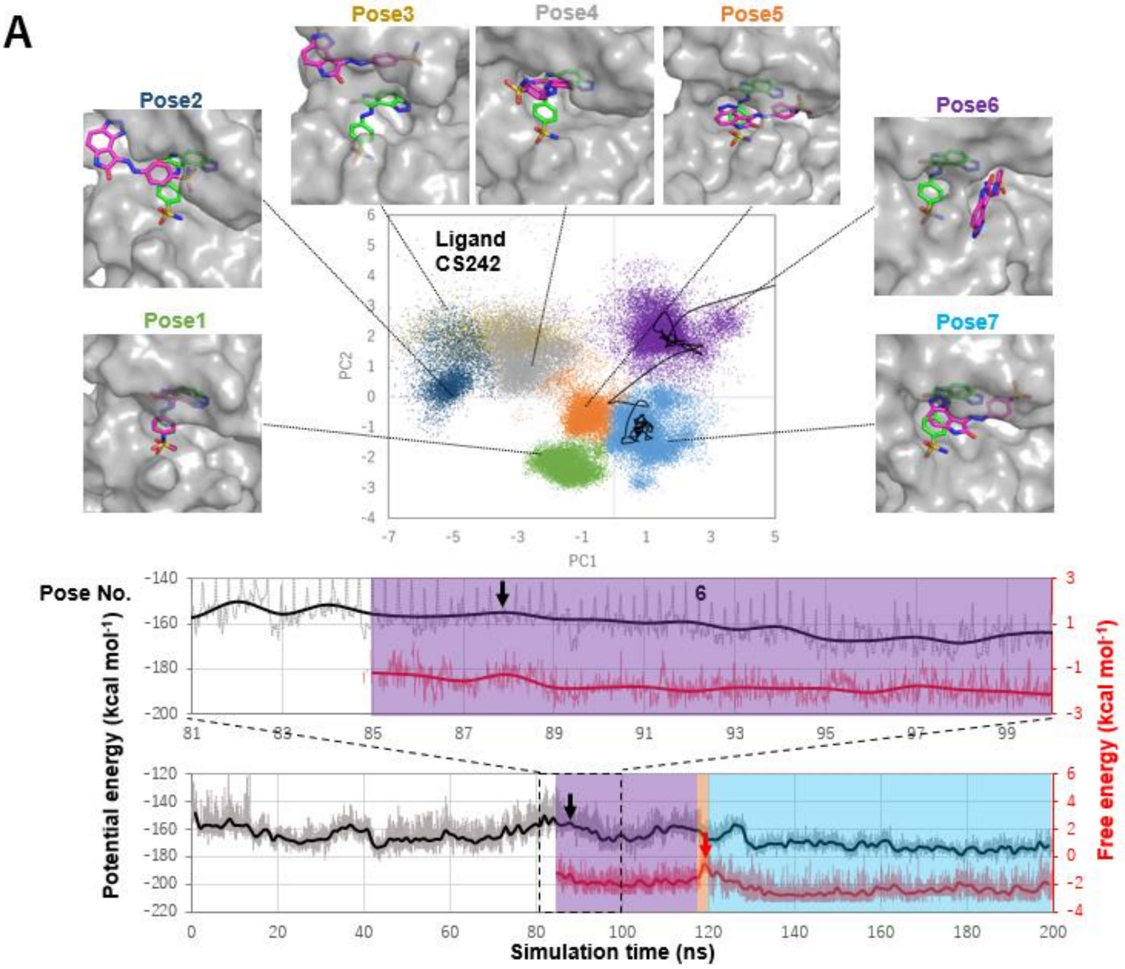

Supplementary Figure 2 (Continued)

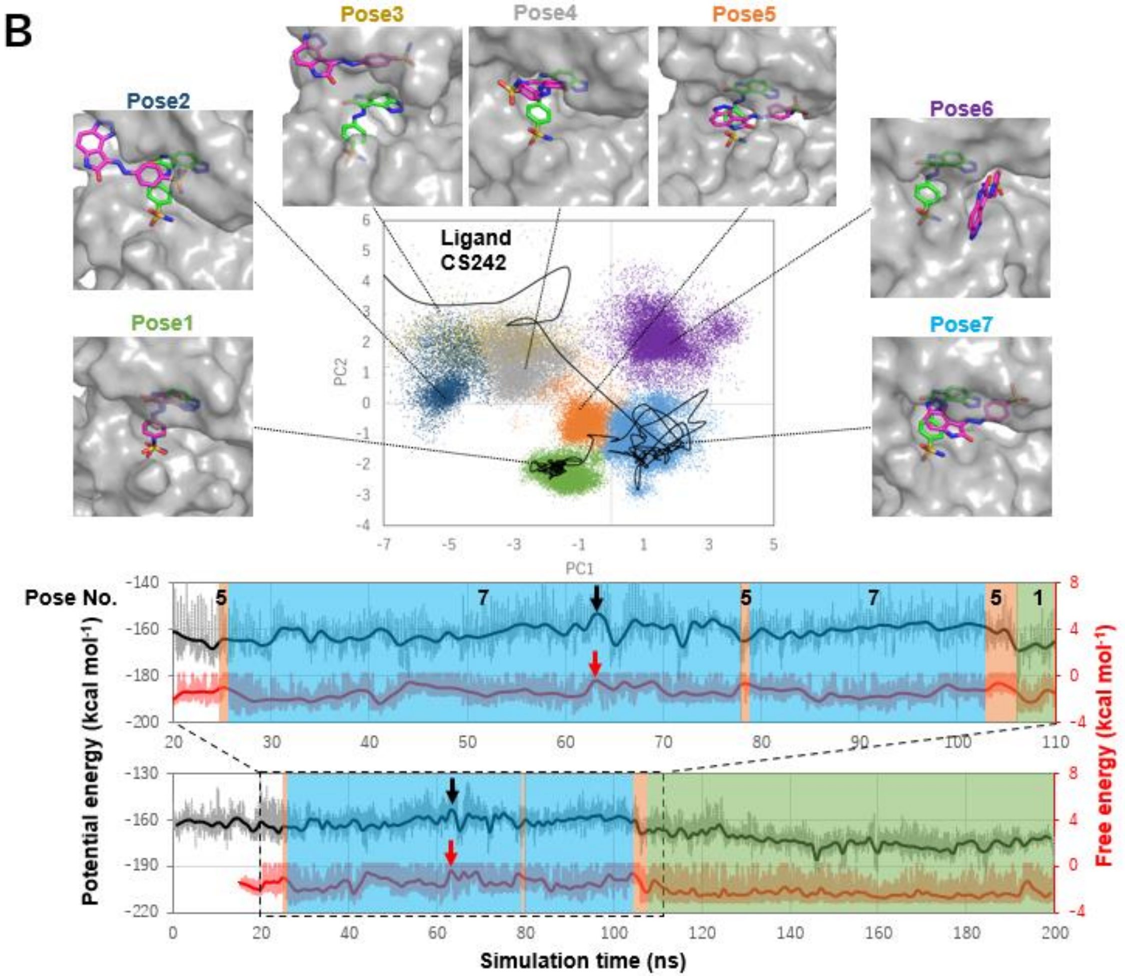

Supplementary Figure 2 (Continued)

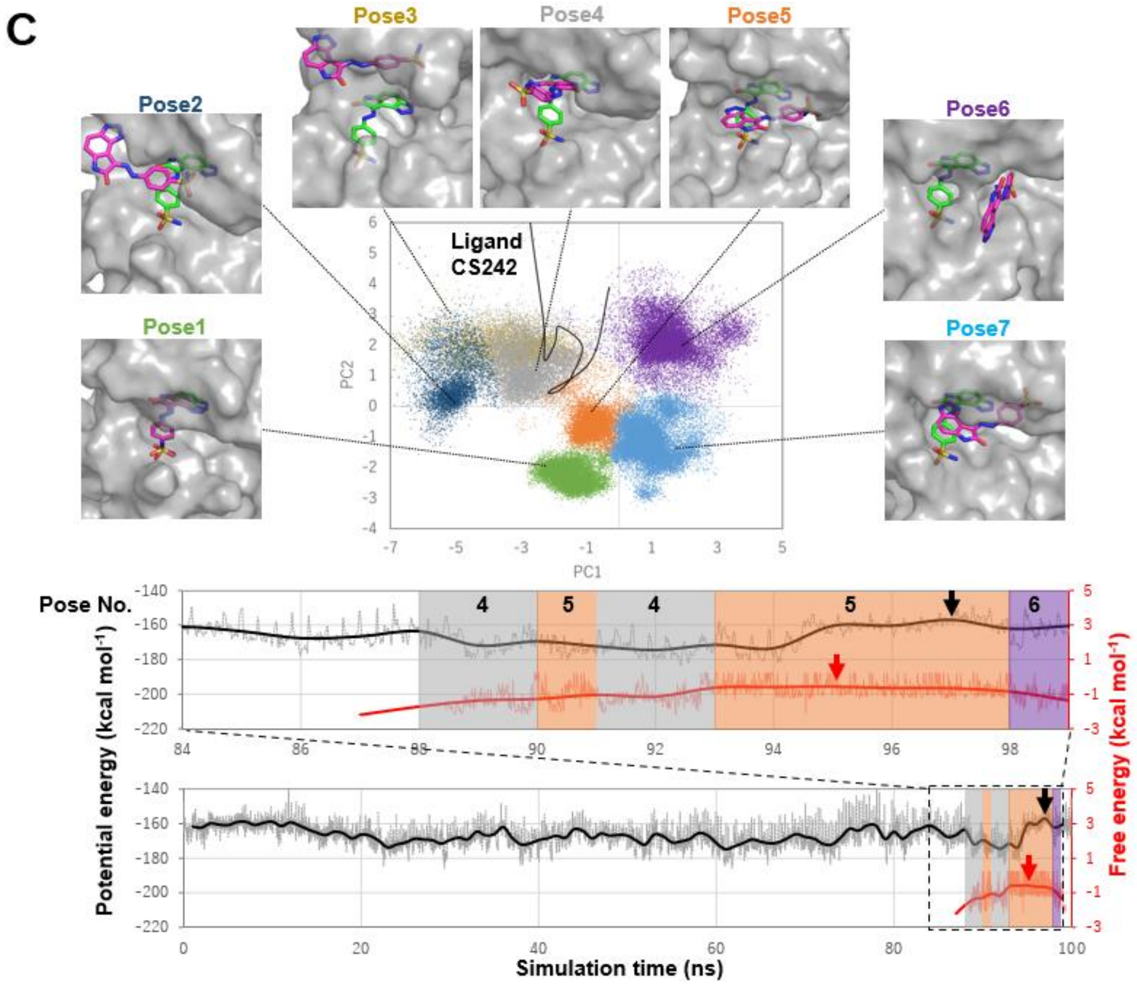

Supplementary Figure 2. Three representative binding pathways of the CS242 ligand to the ATP-binding pocket of CDK2. In pathway (A), the transition state occurs upon entry into the CDK2 pocket; in (B), the transition state is reached during conformational rearrangement in the pocket interior, whereas both ligand binding and unbinding are observed in pathway (C). (top) Projections of binding conformations observed in the whole set of MD trajectories (colored dots) and of a representative binding pathway

(black line) onto the first and second principal components (PC1 and PC2) calculated from PCA (see Methods for details). Seven representative binding poses (magenta sticks) on CDK2 (gray surfaces) are shown along with the crystallographic pose (green sticks), the closest conformation to which was designated as Pose 1. (bottom) Potential energy (black) and free energy (red) trajectories corresponding to the pathway shown in the PCA map. The potential energy was calculated as the sum of the intraligand and intermolecular (protein-ligand and ligand-solvent) contributions. The free energy trajectory was produced from the free energy landscape with respect to (PC1, PC2, and PC3) (Supplementary Fig. 6). The highest-energy transition state is indicated by a black (potential energy) or red (free energy) arrow. An enlarged view of these trajectories close to the highest-energy transition state is also shown in the panel above the whole trajectories. Time intervals in which ligand binding was observed are highlighted in the same color as that used for the binding conformation in the top panel. In 4 out of the 6 binding pathways observed in the hypersound-perturbed MD simulations with N = 50 steps, v_{max} = 400 m/s, and T_{int} = 2,400 N, the binding pose assigned to the highest-energy transition state is the same between potential energy and free energy trajectories.

Supplementary Figure 3

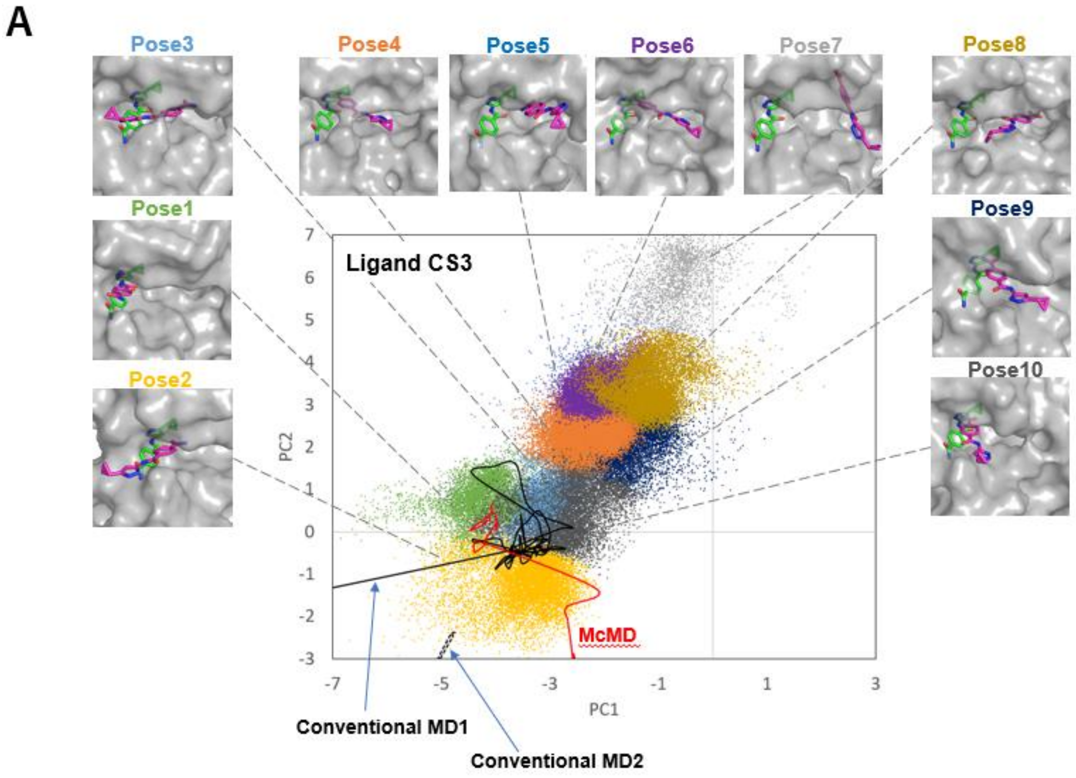

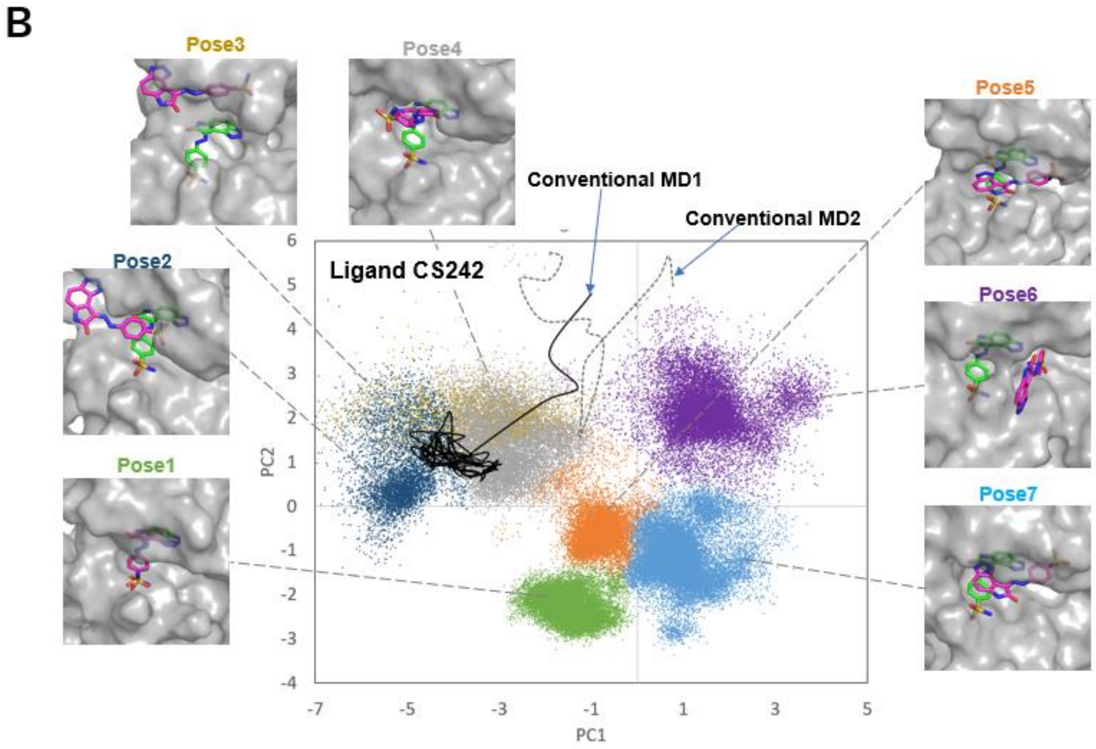

Supplementary Figure 3. Binding pathways of the CS3 (A) and CS242 (B) ligands to the ATP-binding pocket of CDK2 captured by conventional and multicanonical MD (McMD, another type of advanced MD simulations used to efficiently explore protein conformational space [1]. Projections of binding conformations observed in the whole set of simulations (colored dots) and of binding pathways (lines) projected onto the first and second principal components (PC1 and PC2) calculated from PCA (see Methods for details). Two binding pathways (conventional MD1 and MD2) observed in conventional MD simulations are indicated by black solid and dotted lines, while a CS3 binding pathway predicted by McMD [2] is indicated by a red line. Ten (CS3) or seven (CS242) representative binding poses (magenta) are shown along with the crystallographic pose (green), the closest conformation to which was designated as Pose 1.

Supplementary Figure 4

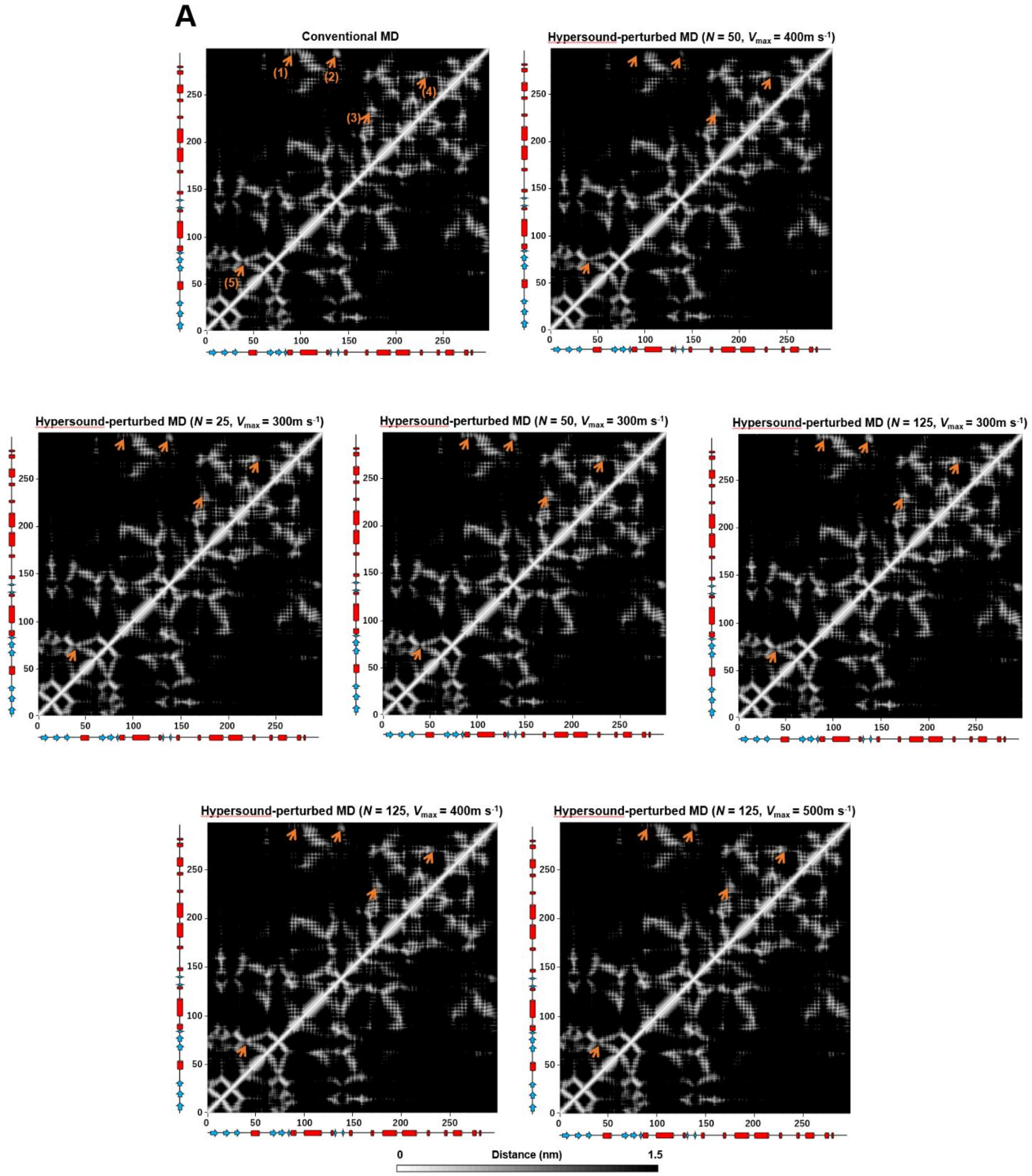

Supplementary Figure 4 (Continued)

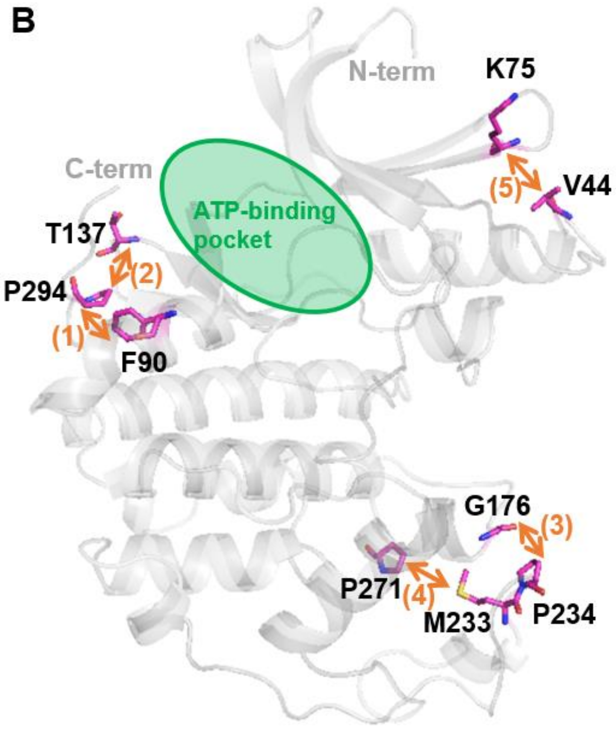

Supplementary Figure 4. Effect of hypersound shock waves on the native structure of the CDK2 kinase. (A) Native contact maps of CDK2 in the presence of CS3, obtained from conventional and hypersound-perturbed MD simulations. The shortest distances between residue pairs were determined using trajectories of 50–100 ns extracted from 10 independent MD simulations of 100 ns without or with hypersound irradiation, where the N and v_{max} values used are indicated at the top of each figure; T_{int} of 2,400N was used across experiments. The inter-residue contacts whose intensities were attenuated by an increase in hypersound frequency (proportional to 1/N) or amplitude (v_{max}) are indicated by orange arrows. (B) Structural location of CDK2 residues affected by hypersound irradiation. Inter-residue contacts corresponding to the numbered arrows in (A) are shown in the native structure of the CDK2 kinase (PDBID: 4EK5), suggesting that hypersound shock waves only perturbed interactions involving the C-terminus or flexible loop regions.

Supplementary Figure 5

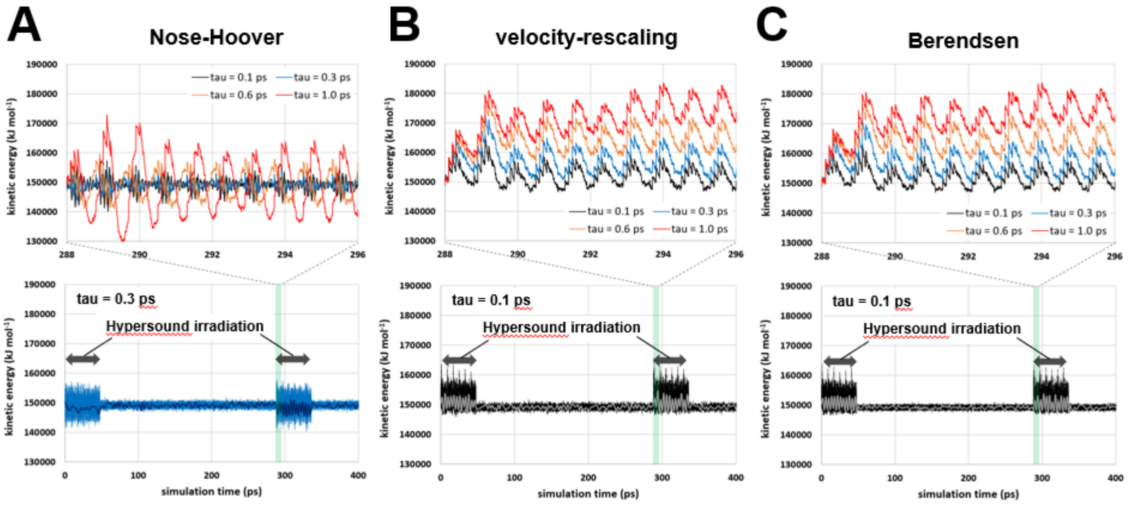

Supplementary Figure 5. Relaxation of the hypersound-induced excess energy using (A) Nose-Hoover, (B) stochastic velocity rescaling, and (C) Berendsen thermostats. Bottom: The total kinetic energy of the liquid water system is plotted every 2 fs (thin lines), and smoothed by a window average of 2 ps (thick lines). A time constant for temperature coupling (τ) is indicated in each panel. The total kinetic energy averaged across the intervals in which hypersound shock waves were generated (indicated by arrows) corresponds to 296 K, 301 K, and 301 K for the plots for Nose-Hoover, stochastic velocity rescaling, and Berendsen thermostats, respectively. Top: An enlarged view of a region ranging from 288 to 296 ps, in which the first shock wave (in the +X direction) in the second series of shock waves was generated, is also shown with the kinetic energy trajectories with different τ values.

Supplementary Figure 6

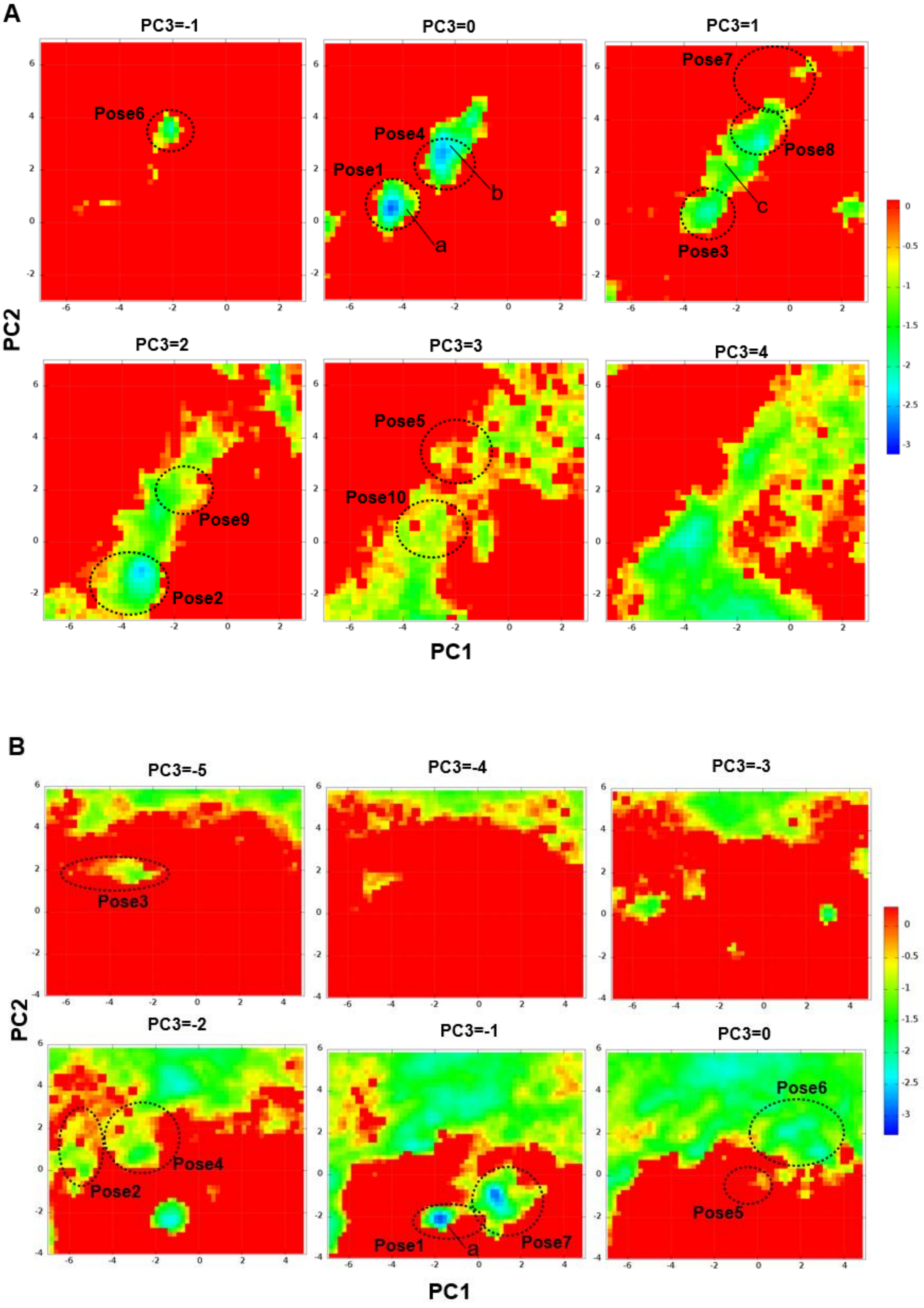

Supplementary Figure 6. The free energy landscapes of (A) CS3 and (B) CS242 binding to the ATP binding pocket of CDK2, which are described as a function of the first three principal components (PC1, PC2, and PC3) calculated from PCA (See Methods), and displayed as 2D slices at different PC3 values. The unit of the free energy is kcal/mol. Each landscape was calculated from the normalized probability distribution, according to $G(\mathrm{PC1, PC2, PC3}) = -RT \ln (P(\mathrm{PC1, PC2, PC3}) / P_{\mathrm{unbound}})$, where R is the gas constant, T is the absolute temperature, and P is the probability density estimated from the hypersound-perturbed MD simulations with N = 50 steps, v_{max} = 400 m/s, and T_{int} = 2,400 N. P_{unbound} is the probability density of the unbound state, and is set to an average density across regions of the PC space in which the ligand is away from the CDK2 surface (>5Å). The 10 (CS3) and 7 (CS242) representative binding poses, which correspond to those shown in Fig. 2A and 2B and Supplementary Fig. 1 and 2, are marked by dotted circles. The locations a-c on the landscape correspond to the crystallographic pose (a) and metastable binding poses estimated by McMD (b and c), which correspond to local minima b and c on Fig. S10A of [2], respectively. The closest conformational cluster to the crystallographic pose (Pose 1) as well as its immediately-preceding state (Pose 4 for CS3 and Pose 7 for CS242) along observed binding pathways (Fig. 2) are successfully assigned to the most stable binding poses. Also, metastable binding poses estimated from the McMD are captured also by the hypersound-perturbed MD simulation, suggesting its high sampling efficiency for ligand binding conformations. The free energy trajectory corresponding to each of 9 (CS3) and 6 (CS242) binding pathways observed in these simulations was produced from the free energy landscapes (Fig. 2A and Supplementary Fig. 1 for CS3 and Fig. 2B and Supplementary Fig. 2 for CS242).

Supplementary Figure 7

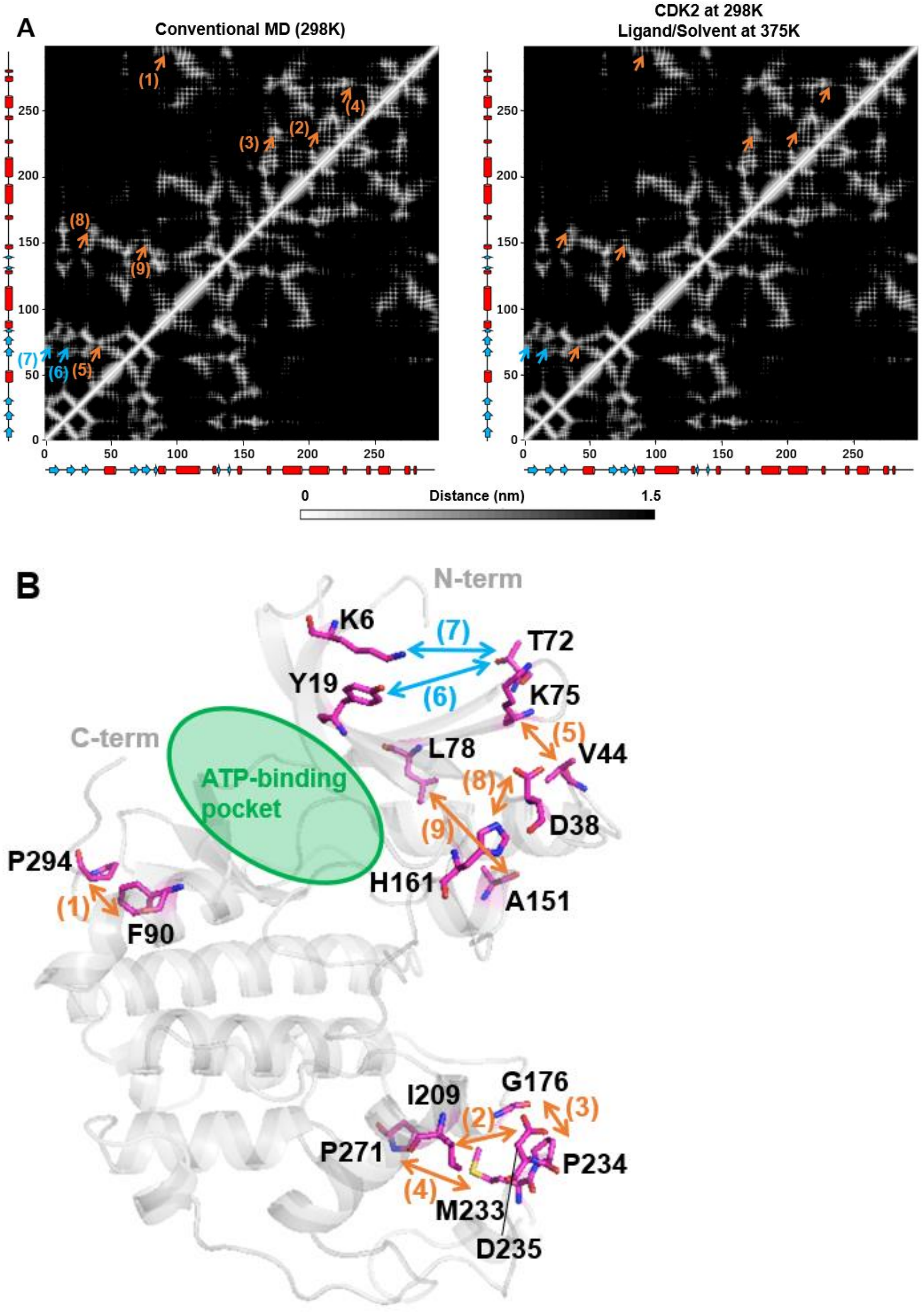

Supplementary Figure 7. Effects of the high ligand/solvent temperature on the native structure of the CDK2 kinase. (A) Native contact maps of CDK2 in the presence of CS3, obtained from conventional MDs at 298 K and high temperature MDs in which the temperature of the ligands and solvent was increased to 375 K while that of the protein was maintained at 298 K. The shortest distances between residue pairs were determined using trajectories of 50–100 ns extracted from 10 independent 100-ns MD simulations. Inter-residue contacts whose intensities were attenuated with an increase in the ligand/solvent temperature are indicated by orange arrows. Non-native contacts generated in the high temperature MD simulation are indicated by cyan arrows. (B) Structural location of CDK2 residues affected by the high temperature MD simulation. Inter-residue contacts corresponding to the numbered arrows in (A) are shown in the native structure of the CDK2 kinase (PDBID: 4EK5), suggesting that the high ligand/solvent temperature not only perturbed interactions involving the C-terminus or flexible loop regions but also induced a partial collapse of rigidly structured regions in the N-lobe of CDK2.

2. Supplementary Tables

Supplementary Table 1. Calculated thermodynamic properties of liquid water

Simulation conditions	Temperature (K)	Diffusion constant (×10^{-5} cm^2 s^{-1})	Kinetic energy (kcal mol^{-1})	Potential energy (kcal mol^{-1})	Total energy (kcal mol^{-1})
Conventional MD at 298 K	298.0 ± 1.2	5.79 ± 0.02	1.78 ± 0.01	-9.61 ± 0.01	-7.83 ± 0.01
Hypersound-perturbed MD at 298 K [a]	295.5 ± 0.1 (297.5 ± 2.2)	6.30 ± 0.10 (5.97 ± 0.06)	1.76 ± 0.00 (1.78 ± 0.01)	-9.77 ± 0.01 (-9.64 ± 0.07)	-8.00 ± 0.01 (-7.86 ± 0.08)
Conventional MD at 305 K	305.0 ± 1.3	6.40 ± 0.07	1.82 ± 0.01	-9.53 ± 0.01	-7.71 ± 0.01
Conventional MD at 313 K	313.0 ± 0.1	7.00 ± 0.02	1.87 ± 0.01	-9.44 ± 0.01	-7.57 ± 0.01
Conventional MD at 328 K	328.0 ± 1.3	8.19 ± 0.02	1.96 ± 0.01	-9.28 ± 0.01	-7.33 ± 0.01

[a] The thermodynamic parameters were calculated using the 0–0.048, 0.288–0.336, 0.576–0.624, 0.864–0.912, 1.152–1.200, 1.440–1.488, 1.728–1.776, 2.016–2.064, 2.304–2.352, 2.592–2.640, 2.880–2.928, 3.168–3.216, 3.456–3.504, 3.744–3.792, 4.032–4.080, 4.320–4.368, 4.608–4.656, and 4.896–4.944 ns trajectories (corresponding to the intervals in which hypersound shock waves were generated), extracted from a 5-ns MD simulation. Averages calculated over the whole 5-ns trajectory, which includes time intervals between shock wave generation (T_{int} of 240 ps in Fig. 1A), are indicated in parentheses.

Supplementary Table 2. Summary of simulations of ligand binding to the ATP pocket of CDK2

ligand	method	number of 100-ns simulations	N [a]	v_{max} (m s^{-1}) [a]	T_{int} [a]	number (percentage) of binding events	number of stable binding events [b]
CS3	Conventional MD	283	-	-	-	2 (0.7%)	1
	Hypersound-perturbed MD	180	25	300	2,400N	22	7
		200	50	300	2,400N	6	1
		177	50	400	2,400N	22 (12.4%)	9
		180	125	300	2,400N	0	0
		200	125	400	2,400N	3	0
		20	125	500	1,440N	3	2
		180	125	500	2,400N	11	5
		total: 1,137				67	24
CS242	Conventional MD	369	-	-	-	2 (0.5%)	1
	Hypersound-perturbed MD	64	50	300	2,400N	2	1
		227	50	400	2,400N	11 (4.8%)	6
		8	125	500	480N	0	0
		59	125	500	1,440N	1	0
		4	125	700	2,400N	0	0
		total: 362				14	7
2AN	Conventional MD	100	-	-	-	2 (2.0%)	0
	Hypersound-perturbed MD	100	50	300	2,400N	8 (8.0%)	1
9YZ	Conventional MD	100	-	-	-	7 (7.0%)	2
	Hypersound-perturbed MD	100	50	400	2,400N	21 (21.0%)	1

[a] The N, v_{max}, and T_{int} parameters are defined in Fig. 1A. N values of 25, 50, and 125 correspond to hypersound frequencies of 1250, 625, and 250 GHz, respectively.

[b] Number of MD trajectories in which the formed protein-ligand complex remained stable until the end of the simulation (100 ns).

Supplementary Table 3. Hypersound parameter dependence of probabilities of observing CS3 binding to the ATP pocket of CDK2 [a]

	v_{max} = 300 (m s^{-1})	v_{max} = 400 (m s^{-1})	v_{max} = 500 (m s^{-1})
N = 25	12.2% (22/180) [b]	N/A [c]	N/A [c]
N = 50	3.0% (6/200)	12.4% (22/177)	N/A [c]
N = 125	0.0% (0/180) [b]	1.5% (3/200)	6.1% (11/180)

[a] The probabilities are extracted from Supplementary Table 2 to show their N or v_{max} dependences, where T_{int} is fixed at 2,400 N. The number of binding events out of the total number of 100-ns MD runs are indicated in parentheses.

[b] Computation speeds of hypersound-perturbed MDs with (N = 25 steps and v_{max} = 300 m/s) and (N = 125 steps and v_{max} = 300 m/s) were 40.6 ± 2.6 and 42.4 ± 2.3. ns/day, respectively, while that of conventional MDs was 45.7 ± 0.9. These MD simulations were performed using seven OpenMP threads on a high-performance computing infrastructure equipped with Intel(R) Xeon(R) CPU E5-2680 v4 and NVIDIA Tesla P100 GPGPUs. The computation speed was estimated from ten independent simulations.

[c] N/A indicates that the probabilities could not be estimated because hypersound-perturbed MD simulations crashed because of the high frequency and/or amplitude of the shock waves.

Supplementary Table 4. Probabilities of observing ligand binding within different CDK2 pockets in conventional and hypersound-perturbed MD simulations [a]

Ligand and simulation type	ATP-binding site	Allosteric site 1	Allosteric site 2	Number of 100-ns simulations
CS3				
Conventional MD	0.7% (2)	0.0% (0)	39.2% (111)	283
Hypersound-perturbed MD [b]	12.4% (22)	2.3% (4)	87.6% (155)	177
CS242				
Conventional MD	0.5% (2)	0.0% (0)	32.2% (119)	369
Hypersound-perturbed MD [b]	4.8% (11)	0.9% (2)	57.7% (131)	227
2AN				
Conventional MD	2.0% (2)	36.0% (36)	99.0% (99)	100
Hypersound-perturbed MD [c]	8.0% (8)	39.0% (39)	100.0% (100)	100
9YZ				
Conventional MD	7.0% (7)	0.0% (0)	95.0% (95)	100
Hypersound-perturbed MD [b]	21.0% (21)	4.0% (4)	100.0% (100)	100

[a] The number of binding events is noted in parentheses. The maximum number of binding events per trajectory was set to one, even if multiple events were observed.

[b] Probabilities were estimated from hypersound-perturbed MDs with N = 50 steps, T_{int} = 2,400 N, and v_{max} = 400 m/s.

[c] Probabilities were estimated from hypersound-perturbed MDs with N = 50 steps, T_{int} = 2,400 N, and v_{max} = 300 m/s.

3. Supplementary References

1. Nakajima N., Nakamura H.& Kidera A. Multicanonical ensemble generated by molecular dynamics simulation for enhanced conforma conformational sampling of peptides. *J. Phys. Chem. B* 1997, **101**(5)**:** 817-824.

2. Bekker G. J., *et al.* Accurate Prediction of Complex Structure and Affinity for a Flexible Protein Receptor and Its Inhibitor. *J. Chem. Theory. Comput.* 2017, **13**(6)**:** 2389-2399.
