# Supplementary material for: Exploring ligand binding pathways on proteins using hypersound-accelerated molecular dynamics
Source: Nat Commun. 2021 May 14;12:2793. doi: 10.1038/s41467-021-23157-1 (PMC8121818; doi:10.1038/s41467-021-23157-1)
Supplement: Supplementary file 3 — Description of Additional Supplementary Files [file 41467_2021_23157_MOESM3_ESM.docx]

**Description of Additional Supplementary Files**

File Name: **Supplementary Movie 1.**

Description: Hypersound-perturbed MD simulation (50 ps) of liquid water. Hypersound shock waves were sequentially generated from each of the *X*_0_, *Y*_0_, *Z*_0_, *X*_1_, *Y*_1_, and *Z*_1_ surfaces every 8 ps. Water molecules are displayed as red sticks.

File Name: **Supplementary Movie 2.**

Description: MD simulation of CDK2-CS3 binding (120 of 200 ns) under hypersound irradiation. CDK2 and CS3 are displayed as surface and stick models, respectively. The corresponding potential energy [combining intraligand and intermolecular (protein-ligand + ligand-solvent) components] trajectory is also shown. The last 80 ns of the trajectory were not included in the movie because no significant conformational changes occurred in the ligand.

File Name: **Supplementary Movie 3.**

Description: MD simulation of CDK2-CS242 binding (120 of 200 ns) under hypersound irradiation. CDK2 and CS242 are represented as surface and stick models, respectively. The corresponding potential energy trajectory [combining intraligand and intermolecular (protein-ligand + ligand-solvent) components] is also shown. The last 80 ns of the trajectory were omitted because no significant conformational changes occurred in the ligand.
