# Supplementary material for: Exploring ligand binding pathways on proteins using hypersound-accelerated molecular dynamics
Source: Nat Commun. 2021 May 14;12:2793. doi: 10.1038/s41467-021-23157-1 (PMC8121818; doi:10.1038/s41467-021-23157-1)
Supplement: Supplementary file 7 — Reporting Summary [file 41467_2021_23157_MOESM7_ESM.pdf]

nature research

Corresponding author(s): Mitsugu Araki
Yasushi Okuno

Last updated by author(s): Apr 5, 2021

Reporting Summary

Nature Research wishes to improve the reproducibility of the work that we publish. This form provides structure for consistency and transparency in reporting. For further information on Nature Research policies, see our Editorial Policies and the Editorial Policy Checklist.

Statistics

For all statistical analyses, confirm that the following items are present in the figure legend, table legend, main text, or Methods section.

n/a	Confirmed	
☐	☒	The exact sample size (*n*) for each experimental group/condition, given as a discrete number and unit of measurement
☐	☒	A statement on whether measurements were taken from distinct samples or whether the same sample was measured repeatedly
☐	☒	The statistical test(s) used AND whether they are one- or two-sided<br>*Only common tests should be described solely by name; describe more complex techniques in the Methods section.*
☒	☐	A description of all covariates tested
☒	☐	A description of any assumptions or corrections, such as tests of normality and adjustment for multiple comparisons
☐	☒	A full description of the statistical parameters including central tendency (e.g. means) or other basic estimates (e.g. regression coefficient) AND variation (e.g. standard deviation) or associated estimates of uncertainty (e.g. confidence intervals)
☐	☒	For null hypothesis testing, the test statistic (e.g. *F*, *t*, *r*) with confidence intervals, effect sizes, degrees of freedom and *P* value noted<br>*Give P values as exact values whenever suitable.*
☒	☐	For Bayesian analysis, information on the choice of priors and Markov chain Monte Carlo settings
☒	☐	For hierarchical and complex designs, identification of the appropriate level for tests and full reporting of outcomes
☒	☐	Estimates of effect sizes (e.g. Cohen's *d*, Pearson's *r*), indicating how they were calculated

Our web collection on statistics for biologists contains articles on many of the points above.

Software and code

Policy information about availability of computer code

Data collection	Molecular Operating Environment (MOE) program version 2016.8 # for modeling of the protein-ligand complexes<br>GAMESS version MAY 2012 (R1) or 14 FEB 2018 (R1) # for calculating electrostatic potentials of ligands<br>AMBER Tools version 12 # for assignment of potential parameters of ligands<br>GROMACS version 4.6.5 # for performing conventional MD simulations<br>GROMACS version 4.6.5_hypersound-perturbed (https://github.com/clinfo/gromacs, see the Code availability section) # for performing hypersound-perturbed MD simulations
Data analysis	GROMACS version 4.6.5 # for analyzing MD simulations<br>Python version 2.7 # for analyzing MD simulations<br>R version 3.1.2 # for conformational clustering of the ligand binding poses<br>PyMOL version 1.8 # for visualization of MD trajectories<br>VMD version 1.9.3 # for visualization of MD trajectories

For manuscripts utilizing custom algorithms or software that are central to the research but not yet described in published literature, software must be made available to editors and reviewers. We strongly encourage code deposition in a community repository (e.g. GitHub). See the Nature Research guidelines for submitting code & software for further information.

Data

Policy information about availability of data

All manuscripts must include a data availability statement. This statement should provide the following information, where applicable:

- Accession codes, unique identifiers, or web links for publicly available datasets
- A list of figures that have associated raw data
- A description of any restrictions on data availability

Molecular dynamics data (the input files, MD trajectories, and processed data) are available in the Biological Structure Model Archive under BSM-00050 (https://bsma.pdbj.org/entry/50). All the other data that support the findings of this study are available from the corresponding authors upon request. There was no restriction on data availability.

Field-specific reporting

Please select the one below that is the best fit for your research. If you are not sure, read the appropriate sections before making your selection.

☒ Life sciences ☐ Behavioural & social sciences ☐ Ecological, evolutionary & environmental sciences

For a reference copy of the document with all sections, see nature.com/documents/nr-reporting-summary-flat.pdf

Life sciences study design

All studies must disclose on these points even when the disclosure is negative.

Sample size	The sample size of CDK2-ligand binding simulations was determined as follows.<br>To accurately estimate the probability of observing the ligand binding event (0.5 to 21 %), a total of 100 to 369 binding simulations were performed (Supplementary Table 2). In addition, we carried out a larger number of binding simulations for CS242 than CS3 because the probability of capturing CS242 binding was expected to be lower than that of CS3, based on their experimental kon values (3.35 × 10^5 and 3.21 × 10^4 (1/Ms) for CS3 and CS242, respectively). Also, since the probability for conventional MDs was expected to be lower than that for hypersound-perturbed MDs, we employed the same or a larger number of conventional than hypersound-perturbed MD simulations to increase the accuracy of the probability.
Data exclusions	There are no data exclusions.
Replication	For each CDK2-ligand system, a total of 100 to 369 independent binding simulations were performed with different atomic velocities.
Randomization	Initial atomic velocities for each binding simulation were generated with random seed, according to a Maxwell distribution at 298K.
Blinding	Blinding is not relevant to this study since all conventional and hypersound-perturbed MD trajectories were automatically analyzed by the same procedures.

Reporting for specific materials, systems and methods

We require information from authors about some types of materials, experimental systems and methods used in many studies. Here, indicate whether each material, system or method listed is relevant to your study. If you are not sure if a list item applies to your research, read the appropriate section before selecting a response.

Materials & experimental systems

n/a	Involved in the study	
☒	☐	Antibodies
☒	☐	Eukaryotic cell lines
☒	☐	Palaeontology and archaeology
☒	☐	Animals and other organisms
☒	☐	Human research participants
☒	☐	Clinical data
☒	☐	Dual use research of concern

Methods

n/a	Involved in the study	
☒	☐	ChIP-seq
☒	☐	Flow cytometry
☒	☐	MRI-based neuroimaging
